# CB11, a novel purine-based PPARɣ ligand, overcomes radio-resistance by regulating ATM signalling and EMT in human non-small-cell lung cancer cells

**DOI:** 10.1038/s41416-020-01088-w

**Published:** 2020-09-22

**Authors:** Tae Woo Kim, Da-Won Hong, Joung Whan Park, Sung Hee Hong

**Affiliations:** 1 Division of Radiation Biomedical Research, Korea Institute of Radiological and Medical Sciences, Seoul, 139-706 Republic of Korea; 2 Laboratory of RNA Cell Biology, Graduate Department of Bioconvergence Science and Technology, Dankook University, Jukjeon-ro 152, Suji-gu, Yongin-si, Gyeonggi-do 16892 Republic of Korea

**Keywords:** Cancer therapy, Lung cancer

## Abstract

**Background:**

Peroxisome proliferator-activated receptor γ (PPARγ) agonists frequently induce cell death in human non-small-cell lung cancer (NSCLC) cells. However, majority of NSCLC patients acquire resistance after cancer therapy, and it is still unclear.

**Methods:**

In this study we investigated the apoptotic mechanism and the anti-cancer effects of a novel purine-based PPARγ agonist, CB11 (8-(2-aminophenyl)-3-butyl-1,6,7-trimethyl-1H-imidazo[2,1-f]purine-2,4(3H,8H)-dione), on human NSCLC cells. CB11 mediates PPARγ-dependent cell death, reactive oxygen species (ROS) production, mitochondrial membrane potential (MMP) collapse, cell cycle arrest, lactate dehydrogenase (LDH) cytotoxicity, and caspase-3 activity in human NSCLC cells.

**Results:**

CB11 causes cell death via ROS-mediated ATM-p53-GADD45α signalling in human NSCLC cells, and diphenyleneiodonium (DPI), an NADPH oxidase inhibitor, decreases cell death by inhibiting CB11-mediated ATM signalling. In a xenograft experiment, CB11 dramatically reduced tumour volume when compared to a control group. Furthermore, CB11 induced cell death by inhibiting epithelial-to-mesenchymal transition (EMT) under radiation exposure in radiation-resistant human NSCLC cells. However, PPARγ deficiency inhibited cell death by blocking the ATM-p53 axis in radiation/CB11-induced radiation-resistant human NSCLC cells.

**Conclusions:**

Taken together, our results suggest that CB11, a novel PPARγ agonist, may be a novel anti-cancer agent, and it could be useful in a therapeutic strategy to overcome radio-resistance in radiation-exposed NSCLC.

## Background

Lung cancer is the most common cause of cancer-related deaths in the world and the second most common cancer in the Republic of Korea. Lung cancer consists of two varieties: small-cell lung cancer (SCLC) and non-small-cell lung cancer (NSCLC).^1^ NSCLC constitutes the majority of lung cancers.^2^ The effectiveness of chemotherapeutic agents is often limited by side effects; thus, the discovery of novel agents with less severe side effects is imperative.

Peroxisome proliferator-activated receptors (PPARs) are a subfamily of nuclear hormone receptors that include receptors for thyroid hormone, retinoic acid, and steroids.^3^ PPAR transcription factors regulate both target gene expression and repression upon ligand binding.^4^ PPARs heterodimerise with retinoic X receptor (RXR) and regulate the transcription of target genes after binding to PPAR response elements (PPREs).^5^ Each PPAR, including PPARs α, β, and γ, is encoded in a separate gene and displays differential tissue distribution.^6^ In the liver, PPARα is highly expressed and activates the oxidation of fatty acids and the detoxification of several xenobiotic compounds.^4,7^ PPARβ is expressed in all human tissues and regulates glucose and lipid metabolism.^8^ In addition to its metabolic and anti-inflammatory properties, PPARγ has also been implicated in tumour suppression.^5,9^ PPARγ is highly expressed in many human NSCLC cells, and PPARγ ligands inhibit tumour growth and progression in animal models of lung cancer.^10,11^ Many studies have shown that thiazolidinedione (TZD), a synthetic ligand for PPARγ that include troglitazone, rosiglitazone, pioglitazone, and ciglitazone (Cig), inhibits cancer cell growth.^12^ Rosiglitazone and troglitazone induce G0-G1 cell-cycle arrest and apoptosis in human pituitary tumour cells.^13^ The activation of PPARγ by its ligands can inhibit cell growth by inducing apoptosis and cell cycle arrest in human colon cancer cells.^14^ A combined treatment of PPARγ ligands and γ-radiation synergistically induces apoptotic cell death in human lung cancer cells.^15^ PPARγ-induced apoptosis involves the activation of caspase-3 protease and the inhibition of Bcl-xL/Bcl-2 functions.^16,17^ PPARγ ligand-treated cells show typical features associated with apoptosis such as chromatin condensation and apoptotic bodies.^6^ PPARγ ligands inhibit growth and induce apoptosis in lung cancer cells via alternative mechanisms, which are dependent on the growth conditions and ligands utilised.^18^

Apoptosis is programmed cell death initiated by a death-receptor- or mitochondria-mediated signal.^19,20^ The caspase-cascade signalling system is regulated by several pathways. They include mitochondria-dependent and -independent pathways, an intrinsic pathway involving mitochondrial proteins, a pathway involving BH3-only proteins such as Bax and Bak, and the p53 and cytochrome c release pathway, which stimulates caspase-dependent apoptosis.^21^ Mitochondria play a functional role in apoptosis, and loss of mitochondrial membrane potential (MMP) is known to induce early apoptosis and cytochrome c release.^22,23^

Reactive oxygen species (ROS) are chemically reactive molecules containing oxygen. They include the superoxide anion radical (O2^-^), hydrogen peroxide (H2O2), and the highly reactive hydroxyl radical (OH).^19^ ROS are intracellular messengers for a variety of stimuli and are highly reactive molecules that can cause DNA damage that can lead to cell death via apoptotic mechanisms.^24^ Tumour suppressor protein p53 plays an important role in the apoptotic responses triggered by DNA damage.^25^ DNA damage is recognised by a protein that contains ATM (ataxia telangiectasia mutated) and ATR (ataxia telangiectasia and Rad3-related).^26^ ATM kinase transmits DNA damage signals to p53.^27^ The activated ATM interacts with various downstream substrates such as CHK1, CHK2, and H2AX.^25^ Activation of ATM can lead to cell growth arrest through the actions of Chk1 and Chk2. H2AX controls the recruitment of DNA repair proteins.^28^ Additionally, GADD45α (growth arrest and DNA damage 45) becomes activated after exposure to certain stresses such as ionising radiation (IR), and it is regulated by increased levels of p53.^29^ In response to DNA damage, ATM activates p53, and p53 mediates GADD45α.^30^

Radiation therapy can be highly effective at treating cancer; however, cancer cells can undergo EMT, which induces radio-resistance.^31^ To overcome radio-resistance during cancer therapy, strategies for EMT inhibition are necessary. The EMT process in cancer cells is initiated when E-cadherin expression decreases and N-cadherin increases, indicating a cadherin switch.^32^ Furthermore, vimentin, snail, and slug contribute to the EMT phenotype.^33^ Recent reports suggest that ROS blocks EMT by killing EMT-type cells, and an ROS regulator may be vital in inhibiting radio- or chemo-resistance caused by EMT.^34,35^

In this study, we evaluated the effects of a novel PPARγ agonist candidate, CB11, on apoptosis and investigated the mechanisms underlying these effects in NSCLC cells exposed and not exposed to radiation. Taken together, our results demonstrate that CB11 causes apoptotic cell death via ROS-induced DNA damage signalling (via the ATM-p53-GADD45α axis) in NSCLC cells, and mitochondrial dysfunction contributes to apoptosis by activating caspase-3 and -9. Both CB11 and radiation overcome radio-resistance by inhibiting the EMT phenotype, and it induces apoptotic cell death by regulating the ATM axis in radio-resistant NSCLC cells.

## Methods

### Materials

Propidium iodide (PI), diphenyleneiodonium (DPI), and antioxidants-including N-acetyl cysteine (NAC) were purchased from Sigma-Aldrich (St. Louis, MO). Ciglitazone and the selective PPARγ antagonist GW9662 were obtained from Cayman (Ann Arbor, MI). The PPAR agonist candidate CB11 was obtained from ChemBridge. DCF-DA (2′, 7′-dichlorodihydrofluorescein diacetate) was obtained from Invitrogen (Carlsbad, CA). The caspase inhibitor, z-VAD-fmk, the caspase-3 inhibitor, z-DEVD-fmk and the caspase-9 inhibitor, z-LEHD-fmk were obtained from Calbiochem (LaJolla, CA). The ATM inhibitors, KU60019 and KU55933, were purchased from calbiochem.

### Cell culture and chemical treatment

Human non-small cell lung cancer cell lines (A549 and H460) and pre-adipose mouse cell lines (3T3-L1) were purchased from the American Type Culture Collection (ATCC, Manassas, VA). Cells were grown in Dulbecco’s modified Eagle’s medium (DMEM) supplemented with, 10% foetal bovine serum (FBS), 100 U/mL penicillin, and 100 μg/mL streptomycin (Gibco BRL Life Technologies, Gaithersburg, MD, USA) at 37 °C under 5% CO_2_. Stock solutions of Cig (10 mM) and CB11 (50 mM) were prepared in dimethyl sulfoxide (DMSO). Ciglitazone (Cig, 10 μM) or CB11 (30 μM) were added to culture media in the presence or absence of caspase inhibitors or antioxidants. Culture medium containing 0.2% FBS was used when cells were treated with PPARγ ligands.

### Oil Red O staining

3T3-L1 cells were seeded in a 6-well plate at a density of 2 × 10^4^ cells per well in Dulbecco’s modified Eagle’s medium (DMEM) supplemented with 10% foetal bovine serum (FBS), 100 U/mL penicillin, and 100 μg/mL streptomycin. After the 3T3-L1 cells were at 80% confluency for two days, the medium was changed to DMEM containing 10% FBS, 1 µM dexamethasone (Dex, Sigma-Aldrich), 0.5 mM 3-isobutyl-1-methyl-xanthine (IBMX, Sigma-Aldrich), and 5 µg/mL insulin (Sigma-Aldrich), and the cells were incubated for 48 h. After differentiation, the medium was exchanged with DMEM supplemented with 10% FBS and 5 µg/mL insulin twice every 48 h. At day eight, the ability of differentiated cells to accumulate intracellular lipids was assessed by Oil Red O staining. The cells were washed twice with PBS; fixed with a 3.7% formaldehyde solution, which was removed by rinsing twice in PBS; stained with Oil Red O working solution for 30 min at room temperature; and washed three times with deionised distilled water. Cells were then visualised by phase-contrast microscopy (Olympus, Tokyo, Japan), and images were obtained with a digital camera (Camedia C-5060, Japan). The Oil Red O stain was eluted with isopropyl alcohol, and its absorbance was measured at 510 nm.

### Cell survival assay

Following treatment with CB11, cell viability was measured using a WST-1 (4-[3-(4-iodophenyl)-2-(4-nitrophenyl)-2H-5-tetrazolio]-1,3-benzene disulfonate) assay (Roche Applied Science, Indianapolis, IA, USA). For the WST-1 assay, human NSCLC cell lines (A549 and H460) and radio-resistant NSCLC cells (A549R and H460R) were plated in a 96-well plate (SPL) overnight. The absorbance of each well was measured at 450 nm using an ELISA reader (FLUOstar OPTIMA, BMG Labtech Offenburg, Germany).

### LDH assay

Human NSCLC and radio-resistant NSCLC cells were seeded into a 96-well plate with growth medium and incubated overnight. LDH was measured with a kit from Thermo Scientific Pierce. The activity in each supernatant was determined by adding 100 μL of the reaction mixture and incubating the reactions for 30 min in a dark room. The LDH activity was determined by measuring the absorbance of the samples at 490 or 492 nm using the ELISA reader.

### Colony formation assay

Human NSCLC and radio-resistant NSCLC cells were trypsinised, counted, and plated onto 60-mm dishes at a density of 1000 cells/dish. They were then treated as indicated and cultured for 10–12 days to allow for colony formation. The colonies were fixed and stained with 0.5% crystal violet in 50% ethanol; those consisting of >100 cells per plate were counted. The survival fraction was calculated using the following formula: surviving fraction = the number of colonies formed/number of cells seeded X the plating efficiency of the control group.

### Determination of apoptosis

Apoptotic cells were quantified by measuring the sub-G1 DNA content with the propidium iodide method. A549 and H460 cells were plated in a 60 mm plate at 3.0 × 10^5^ cells/plate. After 24 h, the cells were exposed to CB11 (30 μM) for 24 h. The cells were harvested, fixed with cold 70 % ethanol at −20 °C overnight, washed twice with Dulbecco’s PBS (DPBS), and stained with DPBS containing PI (50 µg/mL; Sigma-Aldrich) and RNase A (50 µg/mL; Sigma-Aldrich). The cell suspension was incubated in darkness at room temperature for 30 min. The cells at each sub-G1 portion were measured using a FACScan flow cytometer (Becton Dickinson, Franklin Lakes, NJ), and the data were analysed using CellQuest Pro software (Becton Dickinson).

### Caspase-3 activity assay

Caspase-3 activity was assessed with Caspase-3 fluorometric assay kit according to the manufacturer’s instruction. Briefly, A549 and H460 Cells (2 × 10^5^ cells/well) were seeded and cultured onto a six-well culture plates, and then treated with CB11 (30 μM) for the indicated times. A caspase-3 colorimetric assay kit (BioVision, USA) was used to identify the activated caspase-3 cleavage and determined by measuring the amount of the chromophore p-nitroanilide (pNA) upon cleavage from the substrate using a spectrophotometer at 405 nm (Molecular Devices). The caspase-3 activity assay data represent the mean ± SD of three independent experiments.

### Transfection

Human NSCLC cells and radio-resistant NSCLC cells (2 × 10^5^ cells/well) were seeded and cultured onto a six-well plate and transfected with double-stranded siRNAs (30 nmol/mL) against, ATM (Santa Cruz), p53 (Santa Cruz) and PPARγ (Santa Cruz), for 24 h using Lipofectamine 2000 reagent (Invitrogen, Grand Island, NY) according to the manufacturer’s protocol. For transient transfection with PPRE-luciferase pGL3 vector, 3T3L1 cells (1 × 10^5^ cells/well) were seed onto a 12-well plate and transfected with luciferase pGL3 vector (2 µg, Promega) and the PPRE-luciferase pGL3 vector (2 µg, Promega). Luciferase reporter activity was assessed on a luminometer with a luciferase assay system (Promega, Madison, WI) according to the manufactures’ protocol. The luciferase assay data represent the mean ± SD of three independent experiments.

### Real-time PCR and western blot analyses

Real-time PCR reactions were performed in triplicate for each sample using an ABI Power SYBR Green PCR Master Mix (Applied Biosystems) with primers specific for E-cadherin [5′-GAACGCATTGCCACATACAC-3′ (sense) and 5′-GAATTCGGGCTTGTTGTCAT-3′ (antisense)], slug [(5′-CATGCCTGTCATACCACAAC-3′ (sense) and 5′-GGTGTCAGATGGAGGAGGG-3′ (antisense)], snail [(5′-GAGGCGGTGGCAGACTAG-3′ (sense) and 5′-GACACATCGGTCAGACCAG-3′ (antisense)], N-cadherin [(5′-GGTGGAGGAGAAGAAGACCAG-3′ (sense) and 5′-GGCATCAGGCTCCACAGT-3′ (antisense)], vimentin [(5′-TGTCCAAATCGATGTGGATGTTTC-3′ (sense) and 5′-TTGTACCATTCTTCTGCCTCCTG-3′ (antisense)] on a Roche LightCycler 96 System (Roche). RNA quantity was normalised with primers specific for β-actin [5′-AAGGCCAAC CGCGAGAAGAT-3′ (sense) and 5′-TGATGACCTGGCCGTCAGG-3′ (antisense)], and gene expression was quantified according to the 2^−ΔCt^ method. For Western blot analyses, 3T3-L1, human NSCLC cells, and radio-resistant NSCLC cells were solubilised in radioimmunoprecipitation assay (RIPA) lysis buffer (Bio-rad). The primary antibodies used were as follows: β-actin, p-p53 (Ser315), and GADD45ɑ (Santa Cruz, 1:1000); cleaved caspase-3, cleaved caspase-9, p-ATM (Ser1981), p-Chk2(Thr68), E-cadherin, N-cadherin, slug, snail, and vimentin (CellSignaling, 1:1000); the PPARγ (Proteintech, 1:1000). Primary antibodies were detected using a horseradish peroxidase-conjugated secondary antibody, and the membranes were visualised by enhanced chemiluminescence (ECL) or ECL-plus (Amersham Biosciences, Little Chalfont, Buckinghamshire, UK).

### Measurement of ROS

NSCLC cells were plated in a 60-mm plate at 3.0 × 10^5^ cells/plate. After 24 h, the cells were exposed to CB11 (30 μM) for 24 h. ROS generation was measured after staining with 5-(and-6)-carboxy-2′,7′-dichlorodihydrofluorescein diacetate (DCF-DA; Molecular Probes), which interacts with ROS to form a fluorescent complex. DCF fluorescence was immediately measured by flow cytometry. Additionally, NSCLC cells were exposed to CB11 (30 μM) for 24 h in the absence or presence of 10 mM NAC or 10 μM DPI. Then, the medium was replaced with medium containing 20 μM DCF-DA under low-light conditions. The cells were incubated at 37 °C for 30 min before being washed and resuspended in PBS. Fluorescence was analysed in at least 10,000 cells by flow cytometry (FACSCalibur; Becton Dickinson).

### Measurement of mitochondrial membrane potentials

The loss of mitochondrial membrane potential during apoptosis was examined by monitoring cells after staining with 3, 3′-dihexyloxacarbocyanine (DiOC_6_; Molecular Probes), a lipophilic cationic dye. Briefly, 3.0 × 10^5^ NSCLC cells were plated in a 60 mm plate, exposed to CB11 (30 μM) for 24 h, and labelled with DiOC_6_ (40 nM) for 30 min at 37 °C. The cells were washed twice with PBS and analysed using a FACSCalibur flow cytometer (Becton Dickinson). In some cases, cells were pre-treated for 2 h with 3, 3′-dihexyloxacarbocyanine, an inhibitor of mitochondrial depolarisation, before treatment with CB11. Data were acquired and analysed using the CellQuest Pro software. The percentage of cells showing reduced fluorescence reflects the loss of mitochondrial membrane potential.

### Immunofluorescence

A549 cells were plated on coverslips, fixed with 4% paraformaldehyde for 10 min at 4 °C and permeabilised with 0.5% Triton X-100 for 10 min at room temperature. The coverslips were incubated with primary antibodies against p-p53 and ɣH2AX in humidified chamber overnight at 4 °C, and then incubated with the secondary antibodies such as Alexa-555-conjugated goat anti-rabbit and Alexa-488-conjugated goat anti- mouse (Invitrogen Molecular Probes, Eugene, OR) for 1 h at 37 °C. Cell nuclei was stained with 4′,6′-diamidino-2-phenylindole (DAPI, Sigma). Samples were analysed using confocal fluorescence microscopy (LSM 710, Carl Zeiss, Germany).

### Animals

Female athymic BALB/c nude mice (*nu/nu*) aged 5 weeks were obtained from OrientBio, Inc. (Daejeon, Korea) and were housed in SPF facility and microisolator cages in KIRAMS (Korea Institutes of Radiological and Science). Mice permitted to adjust for 1 week with free access to sterile standard mouse chow (NIH-7 open formula) and water. The mice were maintained randomly at 50 ± 20% humidity and approximately 21 ± 2 °C on a 12-h light–dark cycle (*n* = 6 mice/group). Mice were randomly divided into two groups, a control group (0.1% DMSO) and CB11-treated group (each group, *n* = 6).

All mice experiments were conducted according to the National Institutes of Health guidelines and based on a protocol approved by the Institutional Animal Care and Use Committee of the Korea Institute of Radiological and Medical Sciences.

### Tumour xenograft mouse models

A549 cells were harvested and counted using haemocytometer (Marienfeld, Germany). Then, cultured A549 cells (2.5 × 10^6^) subcutaneously (sc) implanted into the thigh of the right hind leg of 6-week-old mice. CB11 (5 or 10 mg/kg) was administered intraperitoneally (ip) for 4 days. To anesthetise, mice were injected intraperitoneally with Avertin solution before surgery. After surgery, mice were exposed with CO_2_ in CO_2_ chamber and maintain the CO_2_ flow until the animal has stopped breathing.

### Tumour measurement

Two axes of the tumour (*L*, longest axis; *W*, shortest axis) were measured three times per week using Vernier callipers. Tumour volume was calculated as (*L* × *W*^2^)/2 (mm^3^). To investigate the toxicity of CB11, we measured the body weights of mice. CB11 did not cause any changes in body weight during the experiment.

### Irradiation

Ionising radiation exposure (2 Gy) was performed using ^137^Cs as the radiation source (Atomic Energy of Canada, Ltd., Mississauga, ON, Canada). To combination treatment, NSCLC and radio-resistant NSCLC cells were pre-treated with CB11 (30 μM) for 2 h, and then stimulated and incubated for 24 h after 2, 4, or 6 Gy exposure.

### Generation of radio-resistant A549 and H460cell lines

A549 and H460 cells were repeatedly exposed to 2 Gy radiation daily for 3 months. The surviving cells were kept at 40–70% confluency and maintained in media containing 10% FBS. Radio-resistant cells were verified by comparing parental cells using a colony survival assay

### Generation of PPARγ shRNA stable H460 and H460R cell lines

PPARγ short hairpin RNA (shRNA) was obtained from Santa Cruz. To generate stable cell lines, PPARγ shRNA was transfected into H460 and H460R cells with Lipofectamine 2000 (Invitrogen), and after 48 h, 1 µg/ml puromycin (Sigma) was added to the cultures for selection.

### Statistical analysis

All results were confirmed in at least three independent experiments; Student’s *t*-test was used to compare the means of quantitative data between groups, and a *p* value < 0.05 was considered statistically significant.

## Results

### Identification of novel PPARγ ligands, CB11

**Fig. 1:**
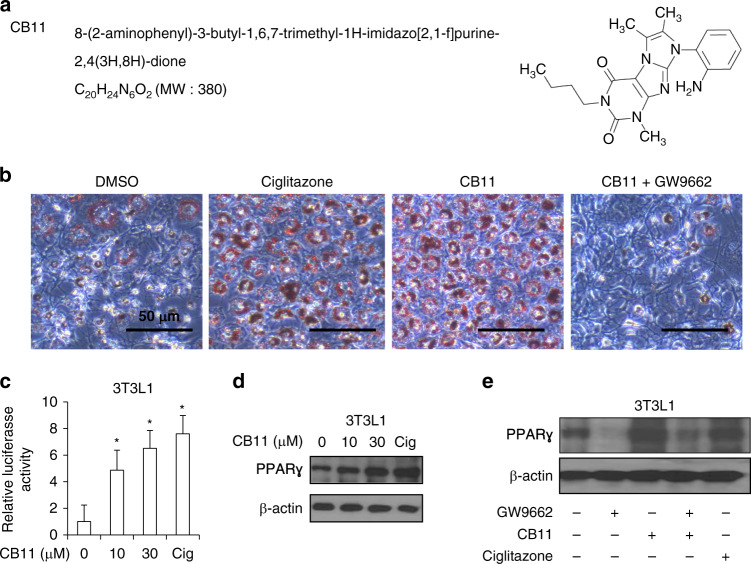
Differentiation of CB-11-treated 3T3-L1 cells into adipocyte-like cells. **a** The chemical structures and molecular formulas of CB11. **b** CB11(30 μΜ)-, Ciglitazone (Cig, 10 μΜ)-, or CB11 (30 μΜ) + GW9662 (20 μM)-mediated adipocyte differentiation was assessed by the presence of Oil Red O-stained droplets. Oil Red O-stained cells were detected using a light microscope by scoring cells from each dish at ×400 magnification. **c** 3T3-L1 cells were transiently transfected with 2 μg of the PPAR response element reporter gene (pGL3-ppre vector) and treated with CB11 or Cig at the doses indicated (CB11, 10 or 30 μΜ; Cig, 10 μΜ); *p < 0.05. **d** Western blot analysis of PPARɣ analysed at the indicated doses in CB11 or Cig-treated 3T3-L1 cells. β-actin was used as a protein loading control. **e** Effect of GW9662 (20 μM) on CB11 (30 μΜ) -treated 3T3-L1 cells. 3T3-L1 cells were pre-treated with GW9662 (20 μΜ) for 4 h and then treated with CB11 for 24 h (30 μΜ). 3T3-L1 cells were also treated with Cig (10 μΜ) as a control. Total lysates were interrogated by Western blot analysis to identify inhibition of PPARɣ. β-actin was used as a protein loading control.

Figure 1a shows the chemical structure of CB11, a novel PPARγ ligand. PPARγ is essential for adipocyte differentiation in that it regulates adipocyte-related genes, including A-FABP and CEBPs.^4,5^ We investigated adipocyte differentiation in 3T3-L1 cells exposed to CB11 and ciglitazone (Cig). CB11-induced adipocyte differentiation was determined by the presence of Oil Red O-stained droplets. As shown in Fig. 1b, 3T3-L1 adipocytes were stained by Oil Red O six days after differentiation by CB11 or Cig. GW9662 is an antagonist of PPARγ, irreversibly inhibits CB11 activity, and blocks differentiation. In the PPRE-luciferase assay, CB11 induces dose-dependent luciferase activity in 3T3-L1 cells when compared with cells exposed to Cig alone (Fig. 1c). To identify the activation of PPARγ, we performed a Western blot analysis. CB11 caused dose-dependent activation of PPARγ, but Cig did not (Fig. 1d). In pharmacological experiments, CB11 and Cig increase PPARγ expression in 3T3-L1 cells, but GW9662 inhibits CB11-mediated PPARγ expression (Fig. 1e).

### CB11 induces apoptotic cell death in NSCLC cells

**Fig. 2:**
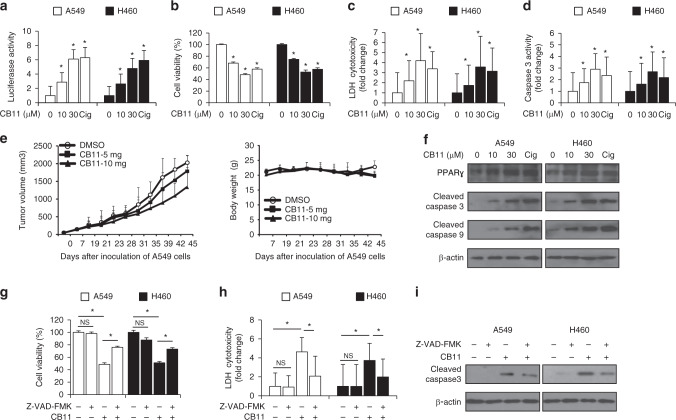
Dose-dependent effects of CB11 on human NSCLC cells (A549 and H460). **a-d** A549 and H460 cells were treated with CB11 or Cig at the doses indicated (CB11, 10 or 30 μΜ; Cig, 10 μΜ). Luciferase activity, cell viability, LDH, and caspase-3 activity assays were performed using this condition; *p < 0.05. **e** A549 cells (2.5 × 10^6^) were implanted (sc) into the thigh on the right hind leg of nude mice (*n* = 6/group). CB11 (5 or 10 mg/kg) or 10% DMSO was administered (ip) once a day for four days. The longest (L) and shortest (W) axes of the tumours were measured, and the tumour volume (mm^3^) was calculated as LW2/2. Body weights of the A549 tumour-xenograft mice were determined twice a week during the experiment. **f** Western blot analyses of PPARɣ, cleaved caspase-3, and cleaved caspase-9 at the indicated doses in CB11 (10 or 30 μΜ, 24 h) or Cig (10  μΜ, 24 h)-treated A549 and H460 cells. β-actin was used as a protein loading control. **g–i** A549 and H460 cells were pre-treated with Z-VAD-FMK (50 μΜ) for 4 h and subsequently treated with CB11 (30 μΜ, 24 h). Cell viability and cell cytotoxicity were determined with WST-1 and LDH assays, respectively; *p < 0.05. Cleaved caspase-3 levels in protein samples were determined by western blot analysis. β-actin was used as a protein loading control.

To probe the PPRE-luciferase activity of CB11 in NSCLC cells, we transfected them with a PPRE-luciferase vector. CB11 caused dose-dependent activation of PPRE-luciferase activity at the dose indicated (Fig. 2a). CB11 and Cig caused a decrease in cell viability and an increase in LDH cytotoxicity and caspase-3 activity (Fig. 2b–d). To examine whether the anti-tumour effect of CB11 could translate to a xenograft mouse model, mice were implanted subcutaneously with A549 cells, and when tumours were palpable, mice were administered 5 or 10 mg/kg of CB11 or DMSO. CB11 treatment inhibited A549 tumour growth in a dose-dependent manner, but the vehicle, alone, did not (Fig. 2e). To investigate the toxicity of CB11, we measured the body weights of the mice. CB11 did not cause any changes in body weight during the experiment (Fig. 2e). Western blot analysis indicated that CB11 (10 or 30 μM, 24 h) and Cig (10 μM, 24 h) up-regulated cleaved caspase-3, and -9 as well as PPARγ in NSCLC cells (Fig. 2f). To study whether CB11-induced apoptotic cell death was inhibited by a pan-caspase inhibitor (Z-VAD-FMK: 50 μM), a caspase-9 inhibitor (Z-LEHD-FMK: 20 μΜ), or a caspase-3 inhibitor (Z-DEVD-FMK: 20 μΜ), we co-treated NSCLC cells with these inhibitors and CB11. This co-treatment inhibited the CB11-induced reduction in cell viability, the increase of caspase-3 and -9 cleavage, and LDH release (Fig. 2g–i and Supplementary Fig. 1A–C).

### CB11 induces cell death via ATM-p53-GADD45α in NSCLC cells

**Fig. 3:**
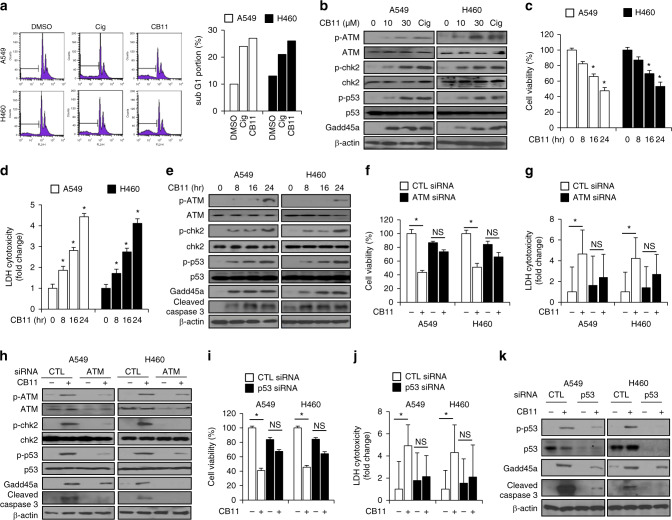
CB11 induces cell death via ATM-p53-GADD45α in NSCLC cells. **a** A549 and H460 cells were treated with CB11(30 μΜ, 24 h) or Cig (10 μΜ, 24 h), and their sub-G1 content was analysed by FACS. **b** Western blot analysis of p-ATM, p-chk2, p-p53, and GADD45α at the doses indicated in CB11(10 or 30 μΜ, 24 h)- or Cig (10 μΜ, 24 h)-treated A549 and H460 cells. β-actin was used as a protein loading control. **c–e** A549 and H460 cells were treated with CB11 for the indicated times (0, 8, 16, and 24); *p < 0.05. **f–k** After A549 and H460 cells were transfected with ATM and p53 siRNA, they were treated with CB11 (30 μΜ). Next, cell viability, LDH, and western blot assays were performed. **f**, **g**, **i**, **j** Cell viability and LDH activity were determined using WST-1 and LDH assays, respectively; *p < 0.05. **h**, **k** Western blot analyses were conducted to study the ATM-p53 pathway in CB11-treated, ATM or p53 knockdown A549 and H460 cells. β-actin was used as a protein loading control.

To examine the mechanism of CB11-mediated cell growth inhibition, cell cycle distribution was evaluated using FACs. NSCLC cells were treated with CB11 (30 μM) or Cig (10 μM) for 24 h. As shown in Fig. 3a, the sub-G1 fraction of cells exposed to CB11 or Cig increased by 2.0- or 2.5-fold, respectively (Fig. 3a). To confirm CB11-mediated cell cycle arrest in NSCLC cells, a Western blot analysis was performed, and the data indicate that CB11 induced dose-dependent phosphorylation of ATR, chk2, and p53 and increased GADD45α levels (Fig. 3b). ATR phosphorylation may contribute to the phosphorylation of chk2 and p53, and following phosphorylation of p53, it may regulate GADD45α in the presence of DNA damage. In NSCLC cells, CB11 mediated a time-dependent decrease of cell viability and an increase of LDH release. Furthermore, Western blot analysis indicated an up-regulation of p-ATM, p-chk2, p-p53, GADD45α, and cleaved caspase-3 (Fig. 3c–e). Immunofluorescence assay showed that CB11 increases p-p53 and ɣH2AX expression, indicating DNA damage, in A549 cells (Supplementary Fig 2A).

### Inhibition of ATM blocks CB11-mediated apoptotic cell death in NSCLC cells

To examine whether CB13 treatment regulates cell death via ATM signalling, we performed pharmacological experiments using ATM inhibitor, such as KU60019 and KU55933. Both KU60019 and KU55933 blocked decrease of cell viability and LDH release in CB11-induced NSCLC cells. Western blot analysis showed that CB11 induced the expression of p-ATM, p-chk2, and cleaved caspase-3 in control NSCLC cells; however, ATM inhibition inhibited CB11-mediated ATM and chk2 phosphorylation and cleavage of caspase-3 (Supplementary Fig. 2B). To further confirm whether CB11 causes apoptotic cell death via the ATM signalling pathway, we performed knockdown experiments using ATM and p53 siRNAs. ATM knockdown suppressed attenuation of cell viability and LDH cytotoxicity in CB11-treated NSCLC cells (Fig. 3f, g). Western blot analysis showed that CB11 induced the expression of p-ATM, p-chk2, p-p53, GADD45α, and cleaved caspase-3 in control NSCLC cells; however, ATM knockdown inhibited CB11-mediated ATM signalling markers and cleavage of caspase-3 (Fig. 3h). We carried out a p53 knockdown experiment in CB11-treated NSCLC cells, and the knockdown prevented the decrease in cell viability and the increase of LDH release caused by CB11 when compared to control cells (Fig. 3i, j). Western blot analysis showed that CB11 caused p-p53, GADD45α, and cleaved caspase-3 up-regulation in control cells, but p53 knockdown suppressed the expression of these proteins by CB11 (Fig. 3k).

### DPI inhibits CB11-mediated apoptotic cell death via ROS in NSCLC cells

**Fig. 4:**
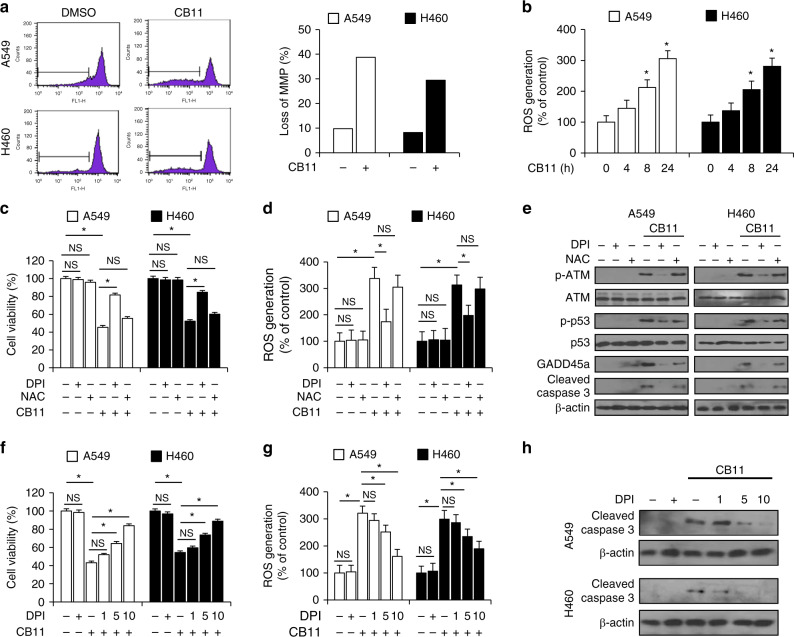
**a** FACs analysis examining mitochondrial membrane potential (MMP) was performed with A549 and H460 cells after CB11 (30 μM) treatment for 24 h and incubation with 50 nM DiOC_6_ for 30 min. **b** A549 and H460 cells were exposed to CB11 (30 μM) for the indicated times, and to measure ROS, these cells were harvested and incubated with the DCFDA fluorescent probe (20 μM) and analysed by FACs. **c–e** A549 and H460 cells were pre-treated with NAC (10 mM) and DPI (10 μM) for 4 h and then treated with CB11 (30 μM) for 24 h. **c**, **d** Cell viability and ROS amounts were determined using a WST-1 assay and incubation with the DCFDA fluorescent probe (20 μM) followed by FACs, respectively; *p < 0.05. **e** Western blot analyses of p-ATM, p-p53, GADD45α, and cleaved caspase-3 derived from DPI, NAC, CB11, DPI + CB11, and NAC + CB11 groups. **f–h** A549 and H460 cells were pre-treated with DPI (1, 5, or 10 μM) for 4 h and then treated with CB11 (30 μM) for 24 h. **f**, **g** Cell viability and ROS amounts were determined using WST-1 assay and incubation with the fluorescent probe DCFDA (20 μM) followed by FACs, respectively; *p < 0.05. **h** Western blot analyses examining the levels of cleaved caspase-3 in CB11-treated A549 and H460 cells (at the doses indicated) in the presence of DPI. β-actin was used as a protein loading control.

Mitochondrial membrane potential (MMP, ΔΨ) plays an important role in mitochondrial apoptotic cell death, and mitochondrial dysfunction confers early apoptotic cell death processes.^36^ To identify whether CB11 induces mitochondrial cell death in NSCLC cells, the loss of MMP during CB11-mediated apoptosis was examined by staining the cells with 3, 3′-dihexyloxacarbocyanine (DiOC_6_). CB11 enhanced the loss of MMP in NSCLC cells (Fig. 4a). According to recent reports, ROS causes cytochrome c to be released from mitochondria and stimulates apoptosis.^37^ To determine whether CB11 mediates ROS production, ROS was quantified using DCFDA, a fluorescent dye. CB11 enhanced ROS release at the indicated times when compared to the DMSO control (Fig. 4b). Furthermore, we introduced ROS inhibitors, including diphenyleneiodonium (DPI, 10 μM) and N-acetylcysteine (NAC, 10 mM), to CB11-treated NSCLC cells. DPI/CB11 treatment inhibited the CB11-mediated decrease in cell viability and ROS production when compared to CB11 treatment, alone, but DPI/NAC treatment did not (Fig. 4c, d). Western blot analysis indicated that DPI/CB11 treatment blocked CB11-mediated p-ATM, p-p53, GADD45α, and cleaved caspase-3 expression in NSCLC cells, but DPI/NAC did not (Fig. 4e). To further confirm whether DPI/CB11 regulates ROS in NSCLC cells, we co-treated them with CB11 (30 μM, 24 h) and DPI (24 h) at the indicated doses. CB11 caused a decrease in cell viability and enhanced ROS production, but DPI blocked these events in a dose-dependent manner (Fig. 4f, g). Western blot analysis indicated that cleaved caspase-3 expression was inhibited in a dose-dependent manner by DPI in CB11-treated NSCLC cells (Fig. 4h).

### CB11 mediates PPARγ-dependent apoptotic cell death in NSCLC cells

**Fig. 5:**
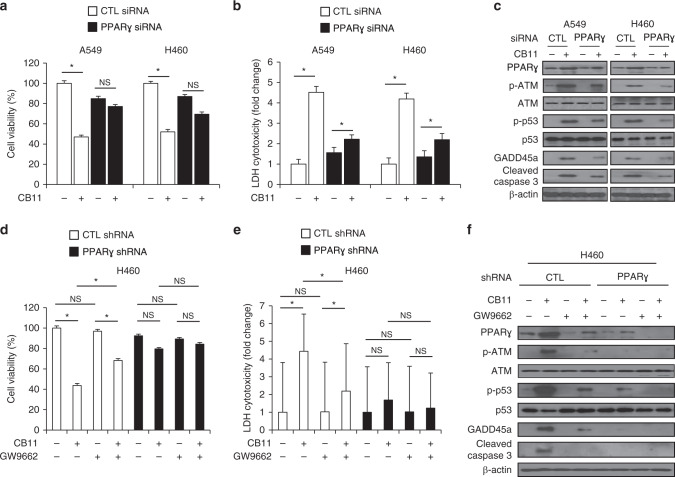
**a–c** After A549 and H460 cells were transfected with PPARɣ siRNA, they were treated with CB11 (30 μΜ), and cell viability and LDH assays were performed along with western blot analyses examining the levels of PPARɣ, p-ATM, p-p53, GADD45α and cleaved caspase-3; *p < 0.05. β-actin was used as a protein loading control. **d–f** PPARɣ shRNA stable H460 cell lines were established by transfection with PPARɣ shRNA, these cells established PPARɣ shRNA. These cells were treated with CB11 (30 μΜ), GW9662 (20 μM), and CB11 + GW9662, and cell viability and LDH assays were performed along with Western blot analyses examining the levels of PPARɣ, p-ATM, p-p53, GADD45α, and cleaved caspase-3; *p < 0.05. β*-*actin was used as a protein loading control.

To confirm whether CB11 induces apoptotic cell death through PPARγ, we treated NSCLC cells with CB11 after transfection with PPARγ-specific siRNA, and cell viability and LDH assays were performed along with Western blot analysis. CB11 caused a reduction in cell viability; an increase in LDH release; and an increase in PPARγ, p-ATM, p-p53, GADD45α, and cleaved caspase-3 expression in control cells. However, PPARγ knockdown inhibited these effects (Fig. 5a–c). To further assess whether PPARγ regulates CB11-induced cell death, we established PPARγ shRNA stable cell lines using H460 cells. Cell viability and LDH assays were performed along with Western blot analysis for both control and PPARγ knockdown H460 cells treated with CB11, GW9662, or CB11/GW9662. CB11 caused a decrease in cell viability; an increase of LDH cytotoxicity; and an up-regulation of PPARγ, p-ATM, p-p53, GADD45α, and cleaved caspase-3 expression in control cells, but CB11/GW9662 inhibited these effects (Fig. 5d–f). Interestingly, PPARγ deficiency suppressed cell viability; the release of LDH; and up-regulation of PPARγ, p-ATM, p-p53, GADD45α, and cleaved caspase-3 in all three groups (Fig. 5d–f).

### Establishment and characterisation of radio-resistant A549R and H460R cells

To assess the cytotoxic effect of CB11 on radio-resistant NSCLC cells, we established A549R and H460R cells. To confirm radio-resistance in A549R and H460R cells, we performed colony formation analyses. Compared to the parental cells, both A549R and H460R cells had larger surviving fraction values, and they increased in a dose-dependent manner (Supplementary Fig. 3A). An increasing number of reports suggest that crosstalk between cancer cells and EMT contributes to radio- and chemo-resistance.^38^ To further characterise the generated A549R and H460R cells, we carried out real-time RT-PCR and Western blot analyses. Real-time RT-PCR data indicated an increase in N-cadherin, vimentin, snail, and slug expression and a reduction in E-cadherin expression when compared to parental cells (Supplementary Fig. 3B). Western blot analysis also showed a decrease in E-cadherin expression in A549R and H460R cells when compared to parental cells (Supplementary Fig. 3C).

### A combination of 2 Gy and CB11 induces apoptotic cell death in radio-resistant A549R and H460R cells

**Fig. 6:**
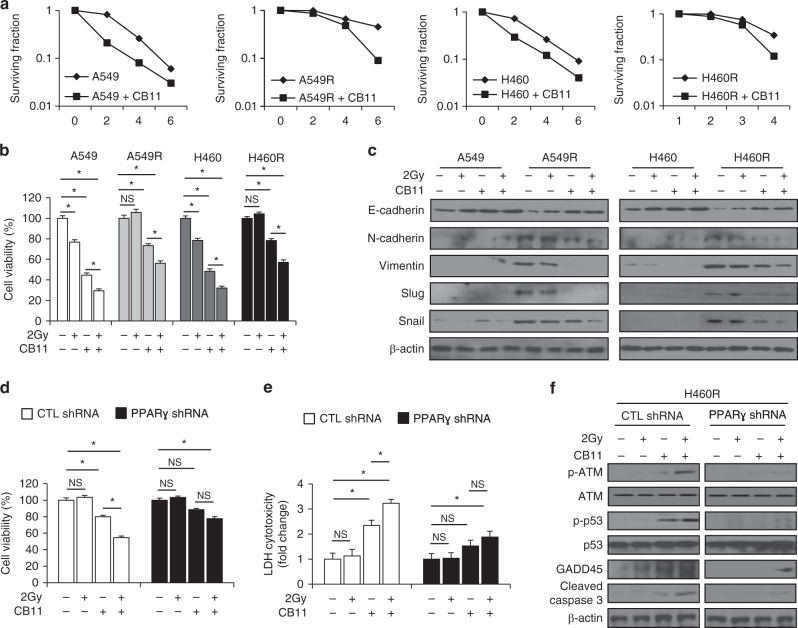
CB11 suppresses the EMT phenotype in radiation-exposed radio-resistant NSCLC cells and induces apoptotic cell death. **a** A clonogenic cell survival assay was conducted with CB11 (30 μΜ, 24 h) treatment after exposure of various radiation doses (0, 2, 4, or 6 Gy). The survival fraction was calculated using the surviving fraction formula in A549, H460, A549R, and H460R cells; *p < 0.05. **b**, **c** A549, H460, A549R, and H460R cells were treated with CB11 (30 μΜ, 24 h) after radiation exposure with 2 Gy. Cell viability was determined for these cells, and Western blot analyses for E-cadherin, N-cadherin, vimentin, slug, and snail were conducted. **d–f** PPARɣ shRNA stable H460R cell lines were established after H460R cells were transfected with PPARɣ shRNA. These cells were exposed to CB11 (30 μΜ), 2 Gy, and CB11 + 2 Gy, and cell viability and LDH assays were performed along with Western blot analyses examining the levels of p-ATM, p-p53, GADD45α and cleaved caspase-3; *p < 0.05. β*-*actin was used as a protein loading control.

To confirm whether CB11 and radiation induce cell death in radio-resistant cells, we performed a colony formation assay. The results showed that CB11 attenuated surviving fraction levels in a dose-dependent manner upon radiation exposure in A549, A549R, H460 and H460R cells. But a greater increase was observed in A549R and H460R cells when compared to A549 and H460 cells (Fig. 6a). These findings indicate that a combination treatment consisting of 2 Gy and CB11 has synergistic cytotoxic effects in radio-resistant NSCLC cells. To probe the related mechanisms of CB11 in radio-resistant NSCLC cells, we performed a cell viability assay. In all, 2 Gy or CB11 treatment caused an ~20% or 50% reduction in cell viability for NSCLC cells, respectively, whereas the combination of 2 Gy and CB11 caused lower cell viability when compared to 2 Gy or CB11 treatment, alone (Fig. 6b). In A549R and H460R cells, 2 Gy exposure did not change cell viability, but CB11 caused an ~20% reduction in the viability of A549R and H460R cells, and the combination of 2 Gy and CB11 caused lower cell viability than 2 Gy or CB11 treatment, alone (Fig. 6b). A combination of 2 Gy and CB11 had greater cytotoxic effects on NSCLC cells when compared to radio-resistant cells; however, 2 Gy/CB11 also had cytotoxic effects in radiation-resistant cells. To further study the underlying mechanism of CB11/radiation treatment, we examined changes in the expression of EMT markers such as E-cadherin, N-cadherin, vimentin, slug, and snail using Western blot analysis. Our data indicated that the three treatment groups, including 2 Gy, CB11, and 2 Gy/CB11, did not change EMT marker expression in NSCLC cells; however, A549R and H460R cells regulated the EMT phenotype, indicating a decrease in E-cadherin and an increase in N-cadherin, vimentin, slug, and snail. Interestingly, CB11 suppresses hallmarks of EMT such as decreased E-cadherin and increased N-cadherin, vimentin, slug, and snail in A549R and H460R cells, and the combination of 2 Gy/CB11 dramatically blocks these changes (Fig. 6c). To further investigate 2 Gy/CB11’s ability to induce apoptotic cell death via PPAR**γ** in radio-resistant NSCLC cells, we established PPARγ knockdown H460R cells. We treated PPARγ knockdown H460R cells with CB11, 2 Gy, and 2 Gy/CB11 and performed cell viability and LDH assays along with Western blot analyses. In all, 2 Gy, alone, had no impact on cell viability, LDH release, or EMT- and ATM-related protein expression in control cells. CB11 treatment, however, caused decreased cell viability; increased LDH release; up-regulation of p-ATM, p-p53, GADD45α, cleaved caspase-3, and E-cadherin; and down-regulation of N-cadherin, vimentin, and slug in control cells (Fig. 6d–f and Supplementary Fig. 4). 2 Gy/CB11 treatment caused lower cell viability; higher LDH cytotoxicity; up-regulation of p-ATM, p-p53, GADD45α, cleaved caspase-3, and E-cadherin; down-regulation of N-cadherin, vimentin, and slug in control cells when compared to CB11 alone (Fig. 6d–f and Supplementary Fig. 4). In PPARγ-deficient H460R cells, 2 Gy had no effect on cell viability, LDH release, the ATM axis, or the expression of EMT-related markers; however, CB11 caused a 10% decrease in cell viability, a 1.5-fold increase in LDH cytotoxicity, and only a weak reduction in slug expression. The combination of 2 Gy/CB11 caused a 20% decrease in cell viability; a 1.8-fold increase in LDH cytotoxicity; weak expression of p-ATM, p-p53, GADD45α, cleaved caspase-3, and E-cadherin; and weak reduction of slug expression, but it did not affect N-cadherin and vimentin (Fig. 6d–f and Supplementary Fig. 4). Taken together, our findings suggest that the combination of 2 Gy/CB11 mediates PPARγ -dependent apoptotic cell death via ATM signalling and EMT in radio-resistant NSCLC cells (Supplementary Fig. 5).

## Discussion

Although the mechanisms underlying the anti-cancer effects of PPARγ agonists remain elusive, many PPARγ ligands are used as therapeutic agents against NSCLC.^39^ PPARγ is widely expressed in various tumours, and synthetic PPARγ ligands have been shown to cause growth inhibition and apoptosis in NSCLC cells.^40^ The functions of PPARγ are well known in adipogenesis and insulin sensitivity, and PPARγ ligands can induce adipocyte differentiation in 3T3-L1 cells, but ligands for PPARα and β cannot.^41^ We found that CB11 can cause adipocyte differentiation, which means CB11 is a PPARγ ligand. Our study demonstrated that PPARγ activation by CB11 or Cig induced apoptotic cell death in human NSCLC cells and that ROS generation may be a key mechanism responsible for these apoptotic actions. PPARγ is expressed in various tumour types, such as liposarcoma, human breast cancer, colon cancer, prostate cancer, and lung cancer, and it inhibits cell proliferation in human NSCLC, ovarian cancer, and pancreatic cancer via apoptosis.^42–44^ An increasing number of studies have reported the utility of PPARγ agonists as anti-cancer agents. PPARγ activation by synthetic ligands has proven an effective anti-cancer regimen through the induction and maintenance of a more differentiated state and/or by apoptosis. However, the molecular mechanisms underlying these effects remain to be explored.

In this study, we showed that the activation of CB11 induces apoptotic cell death in human NSCLC cells. These effects were dose-and time-dependent and increased the population of cells in the sub-G1 peak based on FACs results. One report showed that troglitazone induces a sub-G1 (apoptotic) fraction in the HL60 promyelocytic leukaemia cell line.^45^ Also, a previous study demonstrated that PPARγ induces caspase-dependent apoptosis via ERK activation and mitochondrial damage.^46^ Western blot analysis demonstrated that CB11 induced apoptotic cell death by cleaving caspase-3 and -9 and caused mitochondrial cell death via a loss of MMP. Inhibition of caspase by caspase inhibitors blocked CB11-induced apoptotic cell death in NSCLC cells. Synthetic and endogenous PPARγ ligands induce apoptosis in human lung cancers, and MMP changes have been directly associated with apoptosis since they inhibit the migratory and invasive capabilities necessary for metastasis.^47^ The increase in MMP collapse indicates that these PPARγ agonists induce apoptosis through a mitochondrial pathway.

PPARγ ligands are also known to generate ROS in human cancer cells.^48^ ROS can cause oxidative damage, and its generation is important in apoptotic tumour cell death induced by various anti-cancer agents.^49^ In this study, CB11 generated ROS in NSCLC cells, and DPI (a Nox inhibitor and ROS scavenger) treatment suppressed CB11-mediated ROS generation and apoptotic cell death via ATM signalling. These results indicate that ROS is required for CB11-induced apoptotic cell death in NSCLC cells. Many chemotherapeutic agents induce apoptosis through ROS-mediated cell damage.^50^ Tiron reduced the intracellular ROS levels and partially reduced apoptosis in human lung cancer cells and melanoma cells.^51^ Troglitazone induces apoptotic cell death via ROS, DNA damage, and mitochondrial dysfunction in human hepatocytes but not NAC.^52^ We found that ROS plays a role in DNA damage-induced cell death in CB11-treated NSCLC cells, and DPI/CB11 co-treatment reduced cell damage.

DNA damage is linked to apoptosis and p53.^53^ The tumour suppressor protein p53 plays an important role in apoptotic responses, and DNA damage is recognised by proteins that contain ATM and ATR.^54^ ATM transmits DNA damage signals to p53, and activated ATM interacts with various downstream substrates such as checkpoint kinases (e.g. CHK1, CHK2) and H2AX (histone 2AX).^55,56^ Activation of ATM by these substrates controls the recruitment of DNA repair proteins.^57^ p53 is a transcription factor for cell cycle-regulating genes including p21 and GADD45α, and GADD45α (known as a DNA damage protein) plays a key role in cell cycle arrest-induced apoptotic cell death.^58^ Our data indicate that CB11 induces apoptotic cell death through the ATM-p53-GADD45α axis in NSCLC cells and radio-resistant NSCLC cells. Our data also indicate that CB11, in combination with radiation, is more potent against radio-sensitisation because it regulates the ATM axis and EMT phenotypes.

Radio-resistance impedes radiotherapy, and it activates several EMT phenotypes such as hypoxia-inducible factor 1 (HIF1), ZEB1, and STAT3, which induces metastasis and leads to radiotherapy failure.^59^ Furthermore, IR induces radio-resistance by activating EMT phenotypes, and many studies report a potential link between EMT and radio-resistance.^60^ According to our data, radio-resistant NSCLC cells induced EMT phenotypes. Radiation (2 Gy) exposure had no inhibitory effects on A549R and H460R cells; however, CB11 suppressed the reduction of E-cadherin expression and up-regulation of N-cadherin, vimentin, snail, and slug expression. The combination of 2 Gy/CB11 blocked the reduction of E-cadherin expression, blocked up-regulation of other EMT-related markers and induced apoptotic cell death via the ROS-ATM axis. Thus, radio-resistance is an important obstacle to radiation therapy, and many radiotherapy sensitisers are needed to overcome radio-resistance in cancer therapy.

In summary, CB11 causes apoptotic cell death through ROS generation, ATM axis activation, mitochondria dysfunction, DNA damage, and cell cycle arrest in NSCLC and radio-resistant NSCLC cells. Also, CB11, in combination with radiation, mediates radio-sensitisation via apoptotic cell death and EMT inhibition and overcomes radio-resistance. Taken together, the data indicate that CB11 may potentially increase the efficacy of various anti-cancer agents and could be used therapeutically to overcome radio-resistance.

## Supplementary information

Supplemental Material

## Data Availability

All data presented in this study are included within the paper and its Supplementary information.
