# Supplementary material for: CB11, a novel purine-based PPARɣ ligand, overcomes radio-resistance by regulating ATM signalling and EMT in human non-small-cell lung cancer cells
Source: Br J Cancer. 2020 Sep 22;123(12):1737–48. doi: 10.1038/s41416-020-01088-w (PMC7723055; doi:10.1038/s41416-020-01088-w)
Supplement: Supplementary file 1 — Supplemental Material [file 41416_2020_1088_MOESM1_ESM.pdf]

Supplemental Material

CB11, a novel purine-based PPARɣ ligand, overcomes radio-resistance by regulating ATM signaling and EMT in human non-small-cell lung cancer cells.

Tae Woo Kim, Da-Won Hong, Joung Whan Park, and Sung Hee Hong*

Supplementary Figure

Supplementary Figure 1.

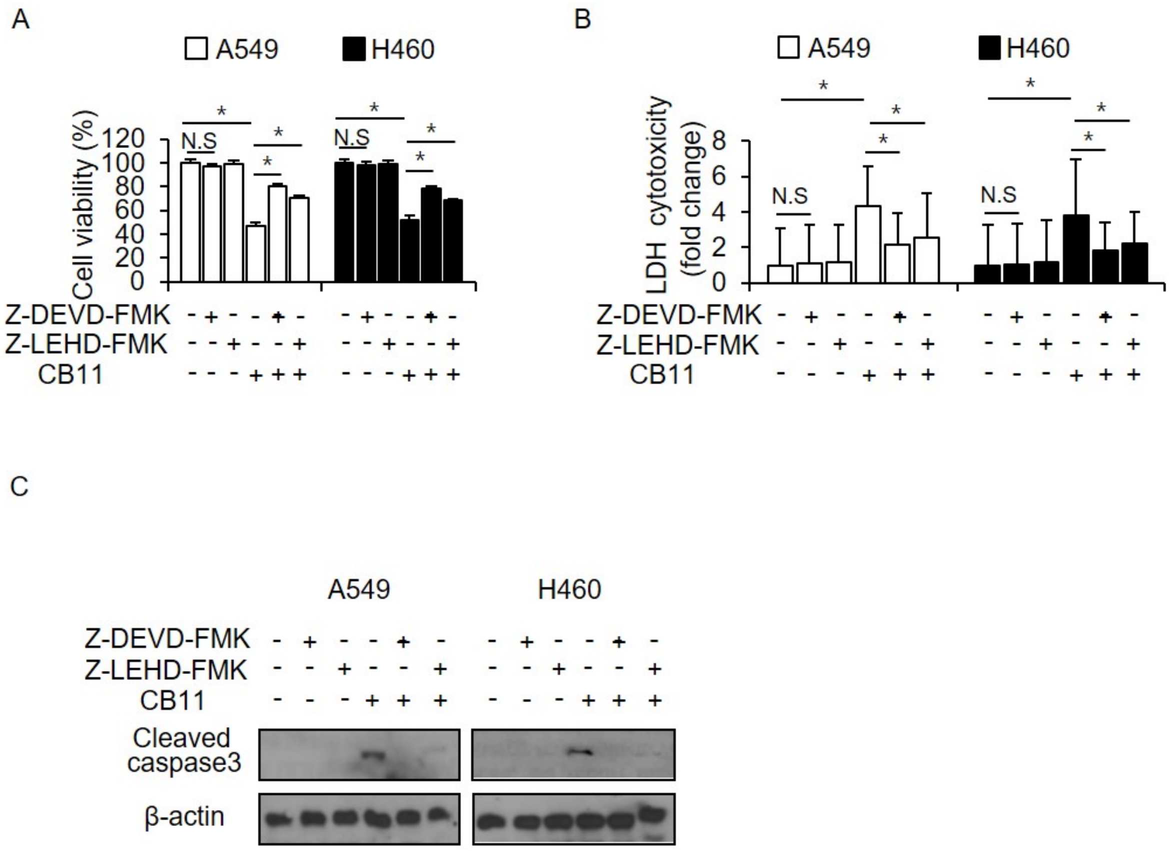

Supplementary Figure 2.

A

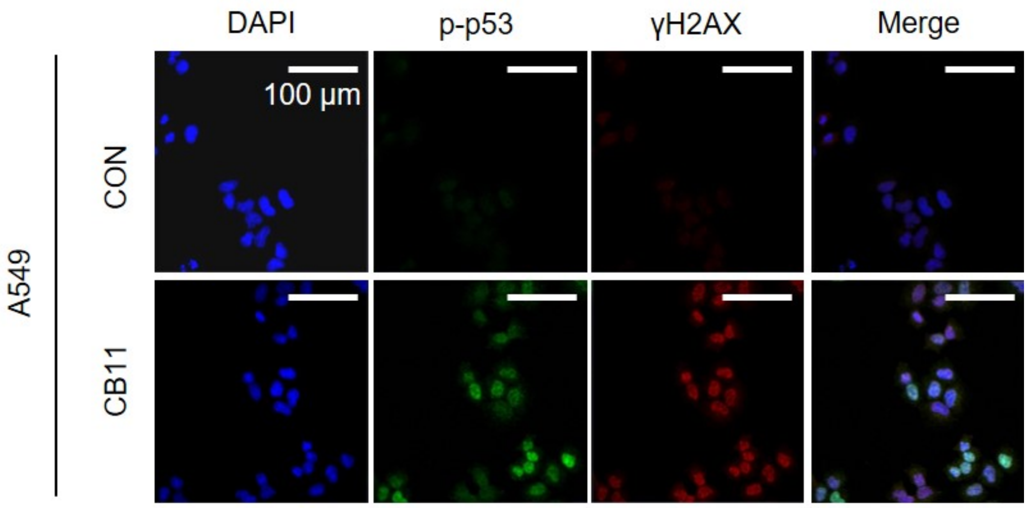

B

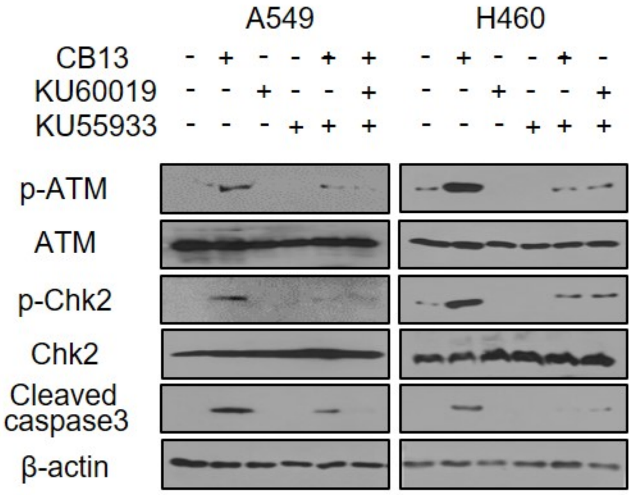

Supplementary Figure 3.

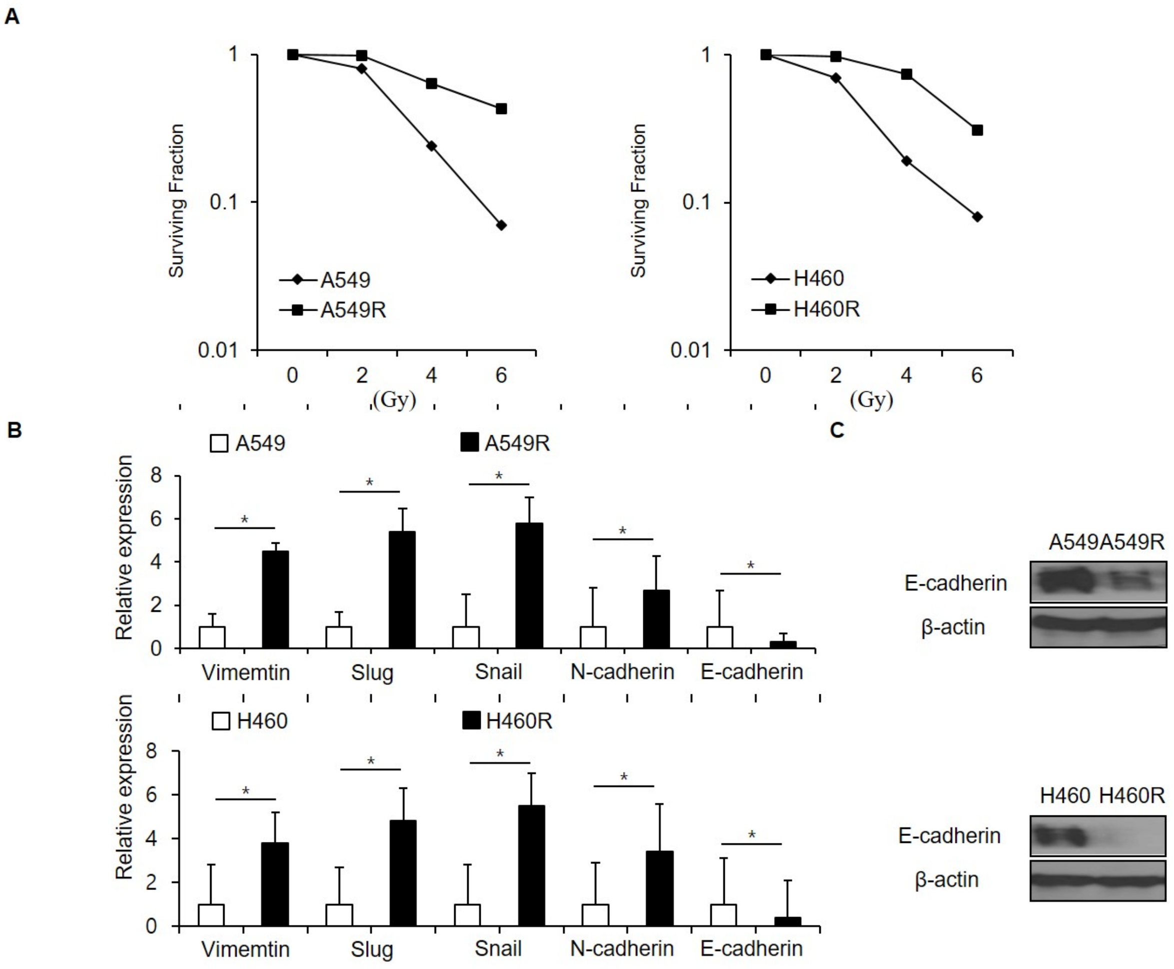

Supplementary Figure 4.

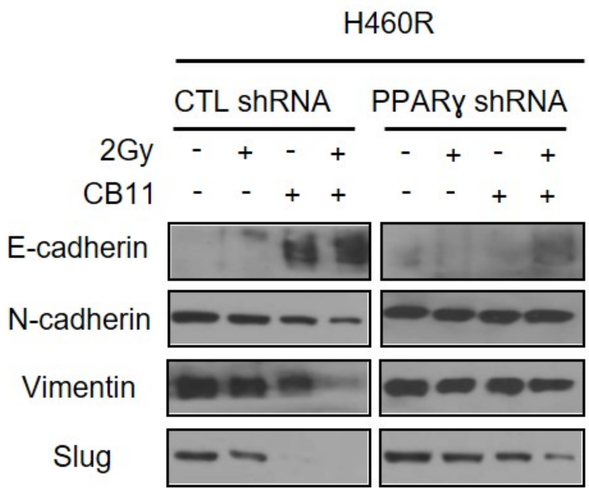

Supplementary Figure 5.

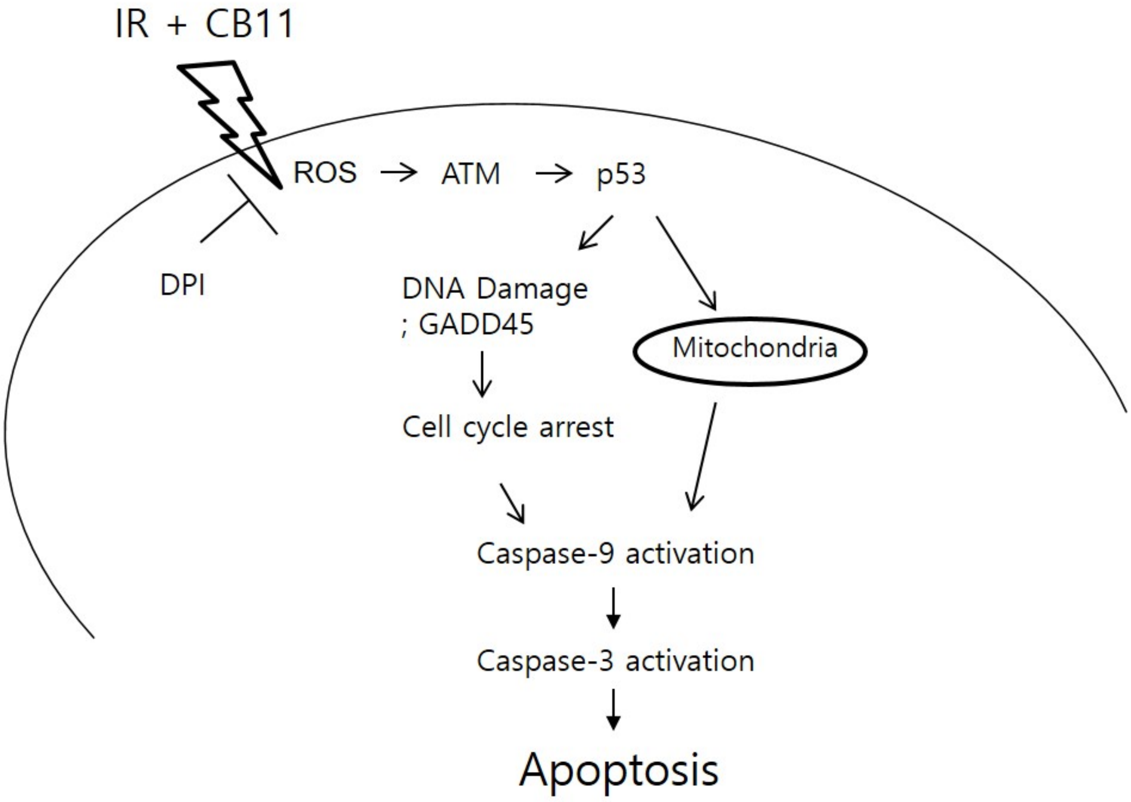

Supplementary Figure Legends

Supplementary Fig 1. The effects of Z-DEVD-FMK and Z-LEHD-FMK on CB11-treated A549 and H460 cells. **A-C** A549 and H460 cells were pretreated with Z-DEVD-FMK (20 μM) or Z-LEHD-FMK (20 μM) for 4 h and subsequently treated with CB11 (30 μM, 24 h). Cell viability and cytotoxicity were determined using WST-1 and LDH assays, respectively; *, $p<0.05$. A Western blot analysis was conducted on protein samples to determine cleaved caspase-3 levels. β-actin was used as a protein loading control.

Supplementary Fig 2. A Analyses of p-p53 and γH2AX responses to CB11. p-p53 and γH2AX confocal immunofluorescence staining in A549 cells treated with CB11 (30 μM, 24 h). p-p53 was labeled in green, γ-H2AX was labeled in red, and nuclei were stained in blue with DAPI (4′,6-diamidino-2-phenylindole). **B** ATM inhibitors suppress CB13-mediated ATM signaling. A549 and H460 cells were treated with or without CB13 (30 μM, 24 h), KU60019 (10 μM, 24 h) and KU55933 (10 μM, 24 h) for 24 h. Western blot analysis was conducted on protein samples to determine p-ATM, ATM, p-chk2, chk2 and cleaved caspase-3 levels. β-actin was used as a protein loading control.

Supplementary Fig 3. Generated radio-resistant A549R and H460R cells acquire an EMT phenotype. **A** A clonogenic cell survival assay was performed with various doses (0, 2, 4, or 6 Gy) of radiation, and the survival fraction was calculated using the surviving fraction formula in A549, A549R, H460, and H460R cells; *, $p<0.05$. **B-C** Real-time RT-PCR was used to detect the expression of E-cadherin, N-cadherin, vimentin, slug, and snail in A549, A549R, H460, and H460R cells, and Western blot analysis was used to detect the protein expression of E-cadherin in A549, A549R, H460, and H460R cells; *, $p<0.05$. β-actin was used as an RNA and protein loading control.

Supplementary Fig 4. PPARɣ shRNA-stable cell lines were established after H460R cells were transfected with PPARɣ shRNA. These cells were treated with CB11 (30 μM) and Western blot analyses were performed to examine the levels of E-cadherin, N-cadherin, vimentin, and slug proteins; *, $p<0.05$. β-actin was used as a protein loading control.

Supplementary Fig 5. Schematic representation of the apoptotic cell death signaling pathway induced by 2Gy + CB01 in radio-resistant NSCLC cells.
